# Improved weight management using genetic information to personalize a calorie controlled diet

**DOI:** 10.1186/1475-2891-6-29

**Published:** 2007-10-18

**Authors:** Ioannis Arkadianos, Ana M Valdes, Efstathios Marinos, Anna Florou, Rosalynn D Gill, Keith A Grimaldi

**Affiliations:** 1 The Dr Arkadianos Clinic, Messogion Av, Athens, Greece; 2 Twin Research Unit, King's College London, UK; 3 Biomedical Engineering Laboratory, National Technical University of Athens, Greece; 4 Sciona Inc, Boulder, 80302, Colorado, USA

## Abstract

**Background:**

Gene-environment studies demonstrate variability in nutrient requirements depending upon individual variations in genes affecting nutrient metabolism and transport. This study investigated whether the inclusion of genetic information to personalize a patient's diet (nutrigenetics) could improve long term weight management.

**Methods:**

Patients with a history of failures at weight loss were offered a nutrigenetic test screening 24 variants in 19 genes involved in metabolism. 50 patients were in the nutrigenetic group and 43 patients attending the same clinic were selected for comparison using algorithms to match the characteristics: age, sex, frequency of clinical visits and BMI at initial clinic visit. The second group of 43 patients did not receive a nutrigenetic test. BMI reduction at 100 and > 300 days and blood fasting glucose were measured.

**Results:**

After 300 days of follow-up individuals in the nutrigenetic group were more likely to have maintained some weight loss (73%) than those in the comparison group (32%), resulting in an age and gender adjusted OR of 5.74 (95% CI 1.74–22.52). Average BMI reduction in the nutrigenetic group was 1.93 kg/m^2^(5.6% loss) vs. an average BMI gain of 0.51 kg/m^2^(2.2% gain) (p < 0.023). Among patients with a starting blood fasting glucose of > 100 mg/dL, 57% (17/30) of the nutrigenetic group but only 25% (4/16) of the non-tested group had levels reduced to < 100 mg/dL after > 90 days of weight management therapy (OR for lowering glucose to < 100 mg/dL due to diet = 1.98 95%CI 1.01, 3.87, p < 0.046).

**Conclusion:**

Addition of nutrigenetically tailored diets resulted in better compliance, longer-term BMI reduction and improvements in blood glucose levels.

## Background

It has been thoroughly documented that the percentage of the population that is overweight and obese is rising to epidemic proportions all over the world with all the attendant health, social and economic consequences [1-5]. While many reasons have been put forward as causes of the epidemic [6] the most likely remain the increased calorie intake and reduced exercise typical of the modern lifestyle. Certainly most people who eat more and exercise less will increase their weight, but arriving at a state of overweight or obesity is a gradual process, taking place over many years of even only slight excess energy intake. For example, in the USA people gain an average of 15 kg (30lb) body weight between the ages of 25 and 55 years old. This level of weight gain represents only ~0.5 kg (1lb) per year, the equivalent of overeating by just a few calories per day [7].

Behavioral treatments can result in a weight loss sufficient to improve health for many patients, but often the weight is regained over time [8]. Although for many people a reduction in weight is difficult to achieve, maintaining the weight loss is even harder. Indeed, few non-surgical treatments for obesity result in sustained weight loss [9]. Long term maintenance of weight loss requires permanent lifestyle changes in exercise and eating habits. These changes need to be significant but not necessarily radical or unachievable if planned over several years of gradual but sustainable weight loss. The National Institutes of Health recommend a 10% weight loss target in the first six months (at a rate of 0.25–0.50 kg/week) followed by a weight maintenance program or further weight loss, at a lower rate, if required [10].

It has long been suspected that "one size does not fit all" in terms of determining the optimal diet for an individual, and this has been demonstrated over the recent years in studies on gene-diet interactions and the emergence of nutrigenetics [11-13]. The goal of nutrigenetics is to add a level of personalization to a prescribed diet, by adjusting it according to genetic variation. For example people carrying the *MTHFR *677T allele require more folate and B vitamins in their diet in order to keep homocysteine levels low [14,15]. Nutrigenetic testing in clinical practice analyzes genes principally involved in the metabolism and transport of nutrients, removal of toxins and protection from oxidation. According to the particular pattern of genetic variation, personalized advice can be generated that contains recommendations on dietary and lifestyle modifications to attain genetically based, specific goals in nutrition and exercise.

The nutrigenetic diet utilized in this study was not designed nor proposed to patients as a weight loss diet; the aim was to optimize the nutrient content of an individual's daily food intake, based on current understanding of an individual's genetic profile. While an individual is achieving weight loss, food consumption is generally reduced and particular nutrients in the diet may not be in adequate supply. Nutrigenetics may be a tool to help achieve optimum nutrient content on an individual basis. Furthermore, the use of nutrigenetics in designing personalized diet and lifestyle programs has the potential to increase motivation and compliance with long-term lifestyle changes.

The Dr Arkadianos clinic in Athens began exploring the use of nutrigenetic testing in weight management protocols in 2003 and initial observations suggested that tailoring the diet according to genetics might improve weight loss and control of biomarkers, such as blood fasting glucose levels. In order to examine these findings in more detail, a formal case history study was initiated. Case histories were followed for a group of 50 patients who took the test and received a personalized diet and compared to a group of 43 patients (matched for age, sex and frequency of clinic visits) who were not tested and who received only the standard clinical diet.

## Methods

Patients with a history of unsuccessful attempts at weight loss (defined as at least two or more unsuccessful attempts) attending a weight management clinic in Athens, Greece were offered nutrigenetic testing. Nutrigenetic kits were used as part of the comprehensive weight management program. The study was constructed through the use of a computerized analysis of patient records. A computer program was written to query the patient clinical records database to select patients who had taken the nutrigenetic test who could be matched for age, sex, starting BMI and number of clinic visits with patients who had not taken the test. In this article, the investigators report the analysis of patient clinical records at a single point in time, which means that different patients were at different time points in their weight management treatment program. The case histories of 50 "nutrigenetic" patients (22 female, 28 male) were compared to those of 43 patients in the non-tested group (18 female and 25 male) which had a follow-up either between 90–365 days (24 nutrigenetic 21 non-tested), a year or more (6 nutrigenetic, 7 non-tested) or both (20 nutrigenetic, 15 non-tested). 7.5 % of study subjects (4 in the nutrigenetic group and 3 in the non tested group) were in the normal weight range (BMI < 25 kg/m^2^). However, they had tried to lose weight on repeated occasions and had failed which is why they attended the clinic.

The study procedure involved periodic analysis of patients' clinical records, which were anonymized and assigned identification numbers. The clinicians involved in the patient treatment were not aware of which patients were included in the study.

All study participants' data was anonymous. Those carrying out the nutrigenetic test signed a consent form and all patient data was handled according to the Greek Code of Medical Deontology and in accordance with the Helsinki Agreement.

### Diet and Exercise

All patients followed a traditional weight management program involving a low glycemic index Mediterranean diet, recommended exercise routines and regular follow-up visits in the clinic (Table 1). The dietary program of the patients in the nutrigenetic group was modified from the standard diet based on the genetic results of each patient. Other than the modifications to the standard diet and exercise program, the patients in both groups were treated in an identical manner

**Table 1:** Sample low saturated fat and low glycemic load Mediterranean diet

**Breakfast:**
One cup of coffee or tea One thin slice of whole grain bread or rye biscuit with one slice of cheese and a slice of turkey ham or with margarine (Becel) and little honey *Or* One portion of cereal with low fat 1.5% milk

**Lunch-Dinner:**

Day 1: One salad of fresh or boiled vegetables, one slice of cheese, one slice of bread. Day 2: *Grilled fish + salad Day 3: *Grilled Chicken + salad Day 4: One portion of green beans, cooked with tomato & olive oil. One slice of cheese and bread Day 5: *Grilled fillet + salad Day 6: One portion of lentils, one slice of cheese, one slice of bread Day 7: *Grilled fish + salad

**Notes :**

• Salads should be dressed with fresh extra virgin olive oil, up to 3 dessert spoons per day • * means that you can eat a lot – but do not overfill • Add a little olive oil to the grilled meat, fish and chicken • You should have one fruit with breakfast, one after dinner and one or two fruits between meals, you may have also one yogurt between meals. • Bread is whole grain or rye. • You may have if you like one glass of wine every day • Program is changed weekly • If increased weight loss is required mainly salads are selected for the dinner meal

### Laboratory Measurements

BMI and blood test results were analyzed from patients' clinical records at regular intervals. A venous blood sample was taken in the early morning after an overnight fast. Serum samples were stored at -40°C until analysis. Fasting glucose was determined using an enzymatic kit (Glucose GOD-PAP, Roche Diagnostic, Germany). Serum total cholesterol and HDL cholesterol concentrations were measured using enzymatic colorimetric methods (CHOL CHOD-PAP, HDL Homegenic Enzymatic reaction, respectively, Roche Diagnostic, Germany).

For nutrigenetic testing, the Sciona MyCellf kit was used (Sciona Inc, Boulder, CO). Cheek cell samples were taken in the clinic using two buccal swabs and the patient completed a comprehensive diet and lifestyle questionnaire. The swabs and samples were sent by courier to Sciona and genetic testing was carried out using a Sequenom Mass Array system. Variants of 19 genes were tested (Table 2).

**Table 2:** Genes and polymorphisms tested in the nutrigenetic patient group.

**Gene**	**Gene symbol**	**Polymorphism**	**% homozygote wild type**	**% heterozygote**	**% homozygote variant**	**HWE p <**
Angiotensin I converting enzyme	***ACE***	INS/DEL	14.6%	48.8%	36.6%	0.99
Apolipoprotein C-III	***APOC3***	3175C>G	73.3%	20.0%	6.7%	0.17
Cystathionine-beta-synthase	***CBS***	699C>T	53.5%	41.9%	4.7%	0.81
Cholesteryl ester transfer protein	***CETP***	279G>A	48.8%	39.5%	11.6%	0.86
Collagen, type I, alpha 1	***COL1A1***	G Sp1 T	58.1%	34.9%	7.0%	0.94
Glutathione S-transferase M1	***GSTM1***	Deletion ^(*)^	52.0%	0.0%	48.0%	N/A
Glutathione S-transferase pi	***GSTP1***	313A>G	57.8%	33.3%	8.9%	0.68
		341C>T	56.8%	34.1%	9.1%	1.00
Glutathione S-transferase theta 1	***GSTT1***	Deletion ^(*)^	86.0%	0.0%	14.0%	N/A
Interleukin 6	***IL6***	-174G>C	66.7%	33.3%	0.0%	0.37
		-634G>C	86.0%	14.0%	0.0%	0.89
Lipoprotein lipase	***LPL***	1595C>G	69.8%	27.9%	2.3%	1.00
5-methyltetrahydrofolate-homocysteine methyltransferase reductase	***MTRR***	66A>G	19.0%	45.2%	35.7%	0.90
5,10-methylenetetrahydrofolate reductase	***MTHFR***	1298A>C	34.0%	48.9%	17.0%	1.00
		677 C>T	48.0%	44.0%	8.0%	0.95
5-methyltetrahydrofolate-homocysteine methyltransferase	***MTR***	2756A>G	59.5%	33.3%	7.1%	0.86
Nitric oxide synthase 3 (endothelial cell)	***NOS3***	894G>T	44.2%	44.2%	11.6%	1.00
Peroxisome proliferator-activated receptor gamma	***PPARG***	Pro12Ala	75.6%	15.6%	8.9%	**0.02**
Superoxide dismutase 2, mitochondrial	***SOD2***	-28C>T	10.0%	54.0%	36.0%	0.57
Superoxide dismutase 3, extracellular	***SOD3***	760C>G	100.0%	0.0%	0.0%	1.00
Tumor necrosis factor	***TNFα***	-308G>A	71.1%	24.4%	4.4%	0.72
Vitamin D receptor	***VDR***	C Taq1 T	23.3%	46.5%	30.2%	0.91
		T Bsm1 C	23.3%	46.5%	30.2%	0.91
		T Fok1 C	11.6%	58.1%	30.2%	0.41

Genotype frequencies in the study group and p-values for Hardy Weinberg Equilibrium (HWE) are shown. (*) the assay only measured presence or absence of the deletion so a HWE test is not applicable.

### Statistical analysis

Baseline characteristics were compared using a one-way analysis of variance in the natural (age, weight, BMI kg/m^2^) or logarithmic scales (glucose, insulin, lipids) or a Pearson's chi-squared test for binary traits. No significant (p > 0.05) deviation from normality was found for the baseline characteristics using a Kolmogorov-Smirnov test of composite normality. Because assumptions of normality are not violated a one-way ANOVA, which is formally equivalent to a t-test, was carried out to test the null hypothesis of no difference in the continuous baseline characteristics between the nutrigenetic and the non-tested group. Change in BMI or weight was compared using analyses of co-variance which included study group (nutrigenetic tested or non-tested) as the independent variable, age and sex as covariates. Odds ratios were estimated using logistic regression models which included age and sex as covariates and belonging to the Nutrigenetic test group (1) or to the non-tested (0) group as the predictor variable. All tests were carried out using S-Plus 6.0 (Insightful Corp, Seattle, WA).

## Results

The genotype frequencies for the genes tested in the nutrigenetic study population are presented in table 2. One of the 24 variants tested deviated significantly from Hardy-Weinberg equilibrium, but given the large number of tests carried out we attribute this observation to type I error. The proportion of patients given personalized advice according to the gene groupings for the individual intervention categories and the rationale for such advice are shown (Table 3). All patients received nutrigenetic based advice in at least one of the intervention categories, with the majority (85%) receiving advice in 4 or more of the 7 possible categories.

**Table 3:** Personalized recommendations given to the Nutrigenetic test patient group in addition to base diet.

**Nutrient intervention group**	**% Receiving modified advice**
**Variation in *MTHFR, MTRR, MTR or CBS***:	**98.6**
*Rationale*: Polymorphisms in genes involved in folic acid metabolism have been shown to influence this pathway affecting plasma homocysteine levels as well as the balance between DNA methylation and synthesis of nucleotides [14, 15].	
*Recommendation*: Add supplement containing 800 mcg folic acid, 15 mg Vitamin B6 and 20 mcg B12	
**Variation in *GSTM1, GSTT1 or GSTP1***:	**76.1**
*Rationale*: Patients with deletions in *GSTM1 *which affect Phase II detoxification processes have been shown to have reduced levels of DNA adducts [16], and increased levels of GSTA1 circulating activity [17], when adequate levels of cruciferous vegetables have been consumed. Risk for lung cancer drops by up to 80% in individuals lacking *GSTM1 *and/or *GSTT1 *genes when consumption of cruciferous vegetables is high [18].	
*Recommendation*: Ensure diet includes regular portions of cruciferous (5 times per week) and allium (daily) vegetables (suggestions and recipes provided to patient). Add broccoli extract and allium supplement if required.	
**Variation in *SOD2, SOD3, NOS3***:	**48.6**
*Rationale*: superoxide dismutase enzymes are free radical scavengers that have important antioxidant activity which can be affected by genetic polymorphism [19]	
*Recommendation*: Add supplements containing antioxidants, Vit A (5,000 IU), Vit C (250 mg) and Vit E (200 IU).	
**Variation in *VDR, COL1A1***:	**87.5**
*Rationale*: Several studies have shown that gene-diet interactions have a role to play in maintenance of bone condition. For example caffeine increased rate of bone loss but only in the presence of the *VDR taq1 *variant [20]. Others have shown gene-diet effects involving calcium [21, 22] and vitamin D [23].	
*Recommendation*: Keep caffeine below 2 cups coffee/day. Increase dairy component of diet (yoghurt, cheese and low fat milk). If required add supplement containing 800 IU vitamin D and 1,300 mg Calcium	
**Variation in *TNFα, IL6, NOS3***:	**65.3**
*Rationale*: Variations in inflammation pathway genes *TNFα *and *IL6 *have been shown to be pro-inflammatory and the effect can be modulated by increased levels of fish oil in the diet [24]	
*Recommendation*: Add supplement Omega 3 (700 – 1,400 mg). Make sure weekly diet contains portions of oily fish	
**Variation in *CETP, LPL, APOC3***:	**79.2**
*Rationale*: Polymorphisms in genes involved in lipid metabolism and transport, in combination with dietary fat intake, have been shown to affect plasma cholesterol levels [25]	
*Recommendation*: The base low fat is already within the limits recommended for these variations so no further specific advice is given but current advice is reinforced and advice given to restrict consumption of dairy foods.	
**Variation in *ACE, PPARG***:	**80.6**
*Rationale*: gene-diet and gene-exercise interactions have been reported to affect blood glucose and insulin levels [26, 27]	
*Recommendation*: The base low glycemic diet is already within the limits recommended for these variations so no further specific advice is given but current advice is reinforced. Extra exercise advised for this group	

The two study groups selected were very similar at the beginning of the clinical program; there were no significant differences in age, sex, BMI, lipids and glucose profiles (Table 4). The majority of the patients were classified as obese with an average BMI of approximately 32 kg/m^2 ^in both groups. No significant difference in co-morbidities was found. In addition to the conditions listed (Table 4), two patients from the nutrigenetic group had had a history of ischemia, two nutrigenetic patients had a history of hypothyroidism and two others had undergone surgical thyroid removal, versus none in the control group. None of these differences were statistically significant.

**Table 4:** Descriptive characteristics of study subjects

	**Non-tested**			**Nutrigenetic patients**			**p-value**
Sample size	43			50			
Gender % female	41.9%			44%			0.99
% obese (BMI = 30 kg/m^2^)	62.8%			70%			0.61
% Hypertension	13.9%			8.0%			0.56

	**mean**	**SD**	**Q1-Q3**	**mean**	**SD**	**Q1-Q3**	

BMI kg/m^2^	33.1	6.6	(29.3–35.8)	33.1	6.7	(29.5–36.9)	0.98
Weight kg	99.1	24.9	(83.6–110.8)	96.5	23.3	(81.7–106.7)	0.60
Age years	45.8	11.5	(37–54.5)	46.0	12.1	(36.5–54.7)	0.92
Glucose mg/dL	94.4	11.5	(87–99)	91.8	22.3	(88–99)	0.65*
Total cholesterol mg/dL	205.8	45.8	(179–235)	214.1	53.0	(191–246)	0.37*
HDL mg/dL	55.6	28.0	(45–61)	50.0	15.8	(40–57)	0.33*
LDL mg/dL	135.0	38.4	(114–157)	137.9	50.1	(111–165)	0.64*
Insulin (mU/L)	11.4	8.0	(5.5–15.2)	13.0	10.3	(6.4–14.3)	0.54*

* analysis of variance carried out on log-transformed variable.

**Figure 1:**
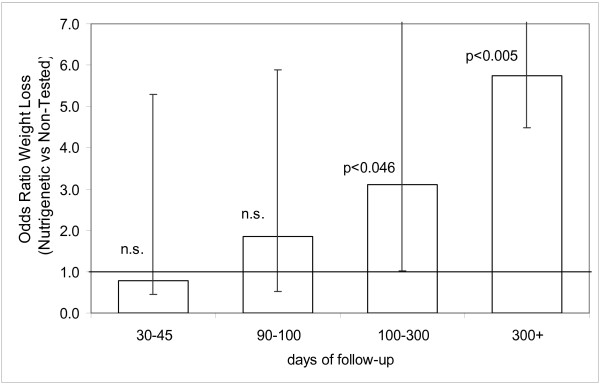
Odds ratio of losing weight (adjusted for age and gender) for individuals in the nutrigenetic test group compared to the control groups. age and sex adjusted odds ratio for weight loss > 0 between the nutrigenetic test group and the non-tested group.

During the first 180 days of weight management therapy, the clinical records demonstrated that the two groups were very similar. Both groups showed a comparable overall average weight loss and approximately 90% had maintained weight reduction (92.9% in the nutrigenetics tested group vs. 88.9% in the non-tested comparison group. Table 5). There was a tendency for the nutrigenetic tested group to have greater BMI reduction, but there were no significant differences up to the 100–300 day period. In the patients who had been followed up for more than 300 days (26 in nutrigenetics tested group, 22 in comparison non-tested group), results were significantly better in the nutrigenetic tested group (p < 0.023). Individuals in the nutrigenetic test group were more likely to have maintained some weight loss (19/26; 73%) than those in the comparison group (7/22; 32%) resulting in an age and gender adjusted odds ratio of 5.74 (95% CI 1.74–22.52 p < 0.005). Average BMI reduction in the nutrigenetic group was 1.93 kg/m^2 ^vs. an average BMI gain of 0.86 kg/m^2 ^(p < 0.023). The difference was more apparent when expressed as a percent of BMI gain/loss, subjects in the nutrigenetic group had a 5.6% loss vs. a 2.2% gain in the non-tested group (p < 0.004). Moreover, from 100 days follow-up onwards, individuals in the nutrigenetic group were significantly more likely to have maintained some weight loss than those in the comparison group (Figure 1). After the 300 days follow-up this resulted in an age and gender adjusted odd raio of 5.74 (95% CI 1.74–22.52).

**Table 5:** Weight and BMI loss (or gain if negative) in the two groups.

	**Non tested group**					**Nutrigenetic group**					P < *
	
**Time point**	**n**	**weight as % of baseline**	**Δ kg**	**Δ BMI (kg/m^2^)**	**% lost weight**	**n**	**weight as % of baseline**	**Δ kg**	**Δ BMI (kg/m^2^)**	**% lost weight**	
baseline	43	100.0%				50	100.0%				
30–45	35	95.4%	4.77	1.59	94.3%	40	96.3%	3.70	2.10	92.5%	0.50
90–100	23	92.2%	8.42	2.78	86.9%	26	93.4%	6.42	3.51	96.1%	0.64
100–300	36	93.4%	6.94	2.35	88.9%	44	92.9%	6.88	3.19	96.4%	0.29
> 300	22	103.2%	-2.74	-0.86	31.8%	26	95.6%	3.61	2.54	73.1%	**0.023**

Weight at follow-up as a percentage of baseline values are shown along with the percentage of individuals in each group who lost weight (regardless of the amount).

* The p-value refers to the analysis of variance comparing the change in BMI between the non-tested and nutrigenetic groups

**Figure 2:**
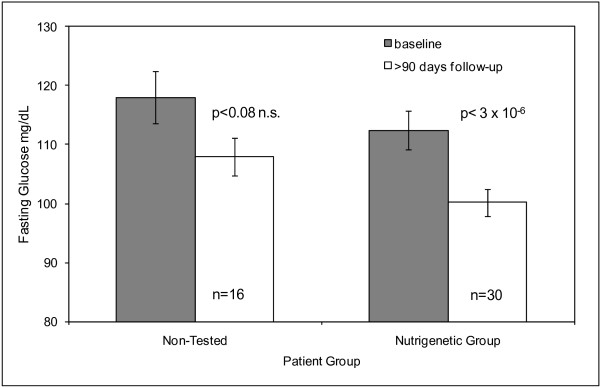
Plasma fasting glucose levels among pre-diabetic subjects at baseline and at or after 90 days follow-up.

Sufficient blood fasting glucose measurement records were available for comparison for a proportion of the patients in the two groups. Among patients with a starting blood fasting glucose above the pre-diabetic level of 100 mg/dL, 57% (17/30) of the nutrigenetic tested group but only 25% (4/16) of the non-tested comparison group had levels reduced to < 100 mg/dL after > 90 days of weight management therapy (odds ratio for lowering glucose to < 100 mg/dL due to diet = 1.98 95%CI 1.01, 3.87, p < 0.046), (Figure 2).

## Discussion

The addition of nutrigenetically tailored diets resulted in better long-term BMI reduction and improvements in blood fasting glucose. Interestingly, the performance of the two groups over the first few months was very similar in terms of weight lost. However, after one year, the non-tested control groups showed a slight average weight gain while the nutrigenetic tested group continued to lose weight, although at a lower rate than during the first 90 days. This suggests that compliance to the weight management programs was better in the nutrigenetics tested group, achieving long term lifestyle changes and resulting in sustained weight loss and improved blood fasting glucose levels. The majority of "pre-diabetic" subjects returned to normal blood fasting glucose levels (< 100 mg/dL), which represents a significant health benefit. We note that the number of pre-diabetic subjects studied in the nutrigenetic group (n = 30) was considerably larger than in the control group (n = 16) which enabled us to detect the improvement in glucose levels in the nutrigenetic group as statistically significant but not in the control group. However, the overall drop in fasting glucose levels was over 20% larger in the nutrigenetic group than in the control group (12.3 mg/dL vs. 10.1 mg/dL). The weight loss recorded in the nutrigenetically tested group after one year was moderate, and it has been well established that even a small weight loss coupled with a healthier diet and lifestyle can lead to significant reduction in risks for diseases associated with excess weight such as diabetes, CVD and metabolic syndrome [4,5].

The nutrigenetic test used in this study determines genetic variation in 19 genes in 7 nutrition intervention groups. The test was not developed specifically as a weight management tool but as a means to optimize and provide a level of personalization to support general healthy eating practices. The gene variants were selected according to documented evidence of gene-diet interactions where a nutrition or exercise intervention has been demonstrated to modify the effect of the variation (see refs cited in Table 3). All patients in the nutrigenetic test group were prescribed a dietary modification in at least one nutrition intervention group with the majority receiving specific advice in four or more groups. Overall, there was considerable variation in the sets of advice given to the individual patients in the nutrigenetic tested group. The differences in long term outcomes between the two study groups suggest that the use of nutrigenetic testing to add personalization to individual diets may be a useful new tool in the management of weight loss and weight control. The maintenance of weight loss is particularly significant in this group of patients who attended the clinic following previous unaided and unsuccessful attempts at weight control. Adding a genetic, personalized component to a weight loss program may improve motivation and compliance, but it is also possible that the personalized diet is better suited by optimizing the content of macro- and micro-nutrients for an individual during a period when overall food consumption is reduced and energy expenditure increased.

We note some limitations to the current study. Our data could be explained by a difference in compliance levels between groups. As there is no placebo arm in this study, it is not possible to evaluate any physiological improvements due to the specific nutritional advice targeted to the patient's genotype. Another limitation is that this study refers only to Caucasian individuals from Greece who had experienced problems losing weight in the past and therefore results may not be necessarily representative of other groups, either from different ethnic or cultural backgrounds, or with different clinical characteristics.

Finally, the sample size, particularly for the comparison of change in glucose levels was fairly modest. However, the effect size seen in the nutrigenetic group was larger than in the non-tested group (0.81 vs 0.66 standard deviations) and a significantly higher proportion of nutrigenetically tested than non-tested individuals lowered their glucose levels to < 100 mg/dL. Therefore, the lower sample size in the non-tested group alone does not explain the difference between the tested and non-tested groups.

The patients in this study group were given a platform diet which consisted of a low-glycemic index Mediterranean balanced diet, with modifications for the tested patients where appropriate. There are a plethora of different types of low calorie diets available to patients who want to lose weight containing very different levels of various macronutrients. Although nutrigenetics is not yet a predictive tool to determine which type of diet will lead to greater weight loss for a particular individual, this is an active area of research. The data from the current study suggest that the use of nutrigenetics to improve and optimize a healthy balanced diet in a clinical setting could be an effective aid in long term lifestyle changes leading to sustained weight loss.

## Competing interests

This work was funded in part by Sciona Inc., Boulder, CO, USA

K. Grimaldi and R. Gill are employees of Sciona Inc

I. Arkadianos is a distributor of Sciona products in Greece

S. Marinos, A.M. Valdes, and A. Florou declare no conflicts of interest.

## Authors' contributions

AMV, who is independent of the sponsors of this project, had full access to all the data in the study and takes responsibility for the integrity of the data and the accuracy of the data analysis.

All authors have read and approved the final manuscript.

*Study concept and design*: IA, RDG, KAG

*Acquisition of data: *IA, EM, AF

*Analysis and interpretation of data: *AMV, KAG, RDG, EM, IA

*Drafting of the manuscript: *KAG, AMV, RDG, IA, AF, EM

*Statistical analysis*: AMV
